# Annotations

**Published:** 1898-03-26

**Authors:** 


### Annotations.

### French Milk.

The announcement that arrangements have been made to place large supplies of French milk upon the English market will be received in this country with mingled feelings. If it has the effect of lowering the present somewhat high price of this commodity, we do not think that the consumer will much object; but there can be but little doubt that the British farmer will be loud in his lamentations, and will crave for protection for his industry. When this question comes to be discussed, as it surely will do, it should be clearly borne in mind that it is the consumer who requires protection rather than the farmer, and that until the latter can show that he is prepared to supply pure milk it is idle for him to ask for foreign milk to be kept out of the country. The statement is made that the milk which is being, and is to be, imported from Normandy, is preserved by the addition of some secret preparation, and that there is no guarantee that the milk may not come from farms the inmates of which are suffering from infectious diseases. These are perfectly valid objections; but, with the case of the Clifton outbreak so recently before us, it is no use our pretending that that milk is inadmissible to our markets unless it has a warranty. As to the use of preservatives, while we would cordially support any measure to abolish the use of them altogether, we cannot admit for a moment that English milk possesses any superiority so far as their presence is concerned. In France the use of chemicals as preservatives of food is prohibited absolutely; Germany and many other countries have also legislated against it; while the fact that some of the largest milk supply companies in London and the provinces entirely forbid it shows that there is no necessity for its use in this country. If the English farmers wish to put a stop to the introduction of foreign milk, let them promote a Bill absolutely prohibiting the use of preservatives. They will then have the market largely to themselves, but to ask for the prohibition of foreign milk on the plea that the ordinary average English milk is of superior purity has about it a dash of the absurd.

### Work for Cripples.

When at music-halls and shows we come across the feats performed by men and women who are destitute of some of their members, we may regard the exhibition as painful; but we cannot avoid admiring the patience and ingenuity which their efforts to overcome their deformity display. Hitherto it does not seem to have occurred to anyone—in this country, at least—to inquire if deformed persons could not be trained to use such limbs as they have, not for exhibition purposes, but for useful work. But it has occurred to a certain Danish clergyman, Pastor Hans Knudsen, of Copenhagen, and the value of the Industrial School for Cripples (Arbeitskole for Vanförer), which he has organised, may be estimated from the fact that the Danish Government subsidises it, "not only because of its humanitarian value, but also because of its practical economic services in lessening the pauper class." Similar schools have been established in all the Scandinavian countries —Norway, Sweden, and Finland—and by the instruction given in them cripples, who elsewhere would be beggars, or dependent on their friends, are enabled to become wholly or partially self-supporting. The cripples are such as are apparently quite helpless—those, as Froken Fleischer, the manager of the [illegible]beitskole of Christiania, explains, "for whom thera-[illegible]ics and surgery can do nothing, and to whom artificial limbs, &c., are for some reason or another of no avail." Some have two fingers instead of ten, and many have none at all. A young girl who possessed no hands managed to weave, sending the shuttle to and fro with the stumps of her arms. Another who was destitute even of arms did embroidery and brush-making with her teeth. The boys do cobbling, brush-making, and light carpentry. It is true that in many cases special contrivances have to be invented for each case to enable the maimed worker to fulfil his task. Things must be clamped to a bench or table when there are no hands to hold them with. A cripples' school of this kind demands from the manager of it continual thought and considerable inventive skill, as well as those moral qualities which are, as a rule, considered the one thing needful in philanthropic work. Even the business faculty, which sets the pupils to work which, with training, they can do as well as ordinary persons, and which will meet with a ready sale, is essential. To many of the pupils the ability to earn their own living is a matter of importance, and in all the moral satisfaction of feeling that they are no longer shut out from the ordinary activities of life is of the highest value. As Froken Fleischer expresses it, "when one of her pupils holds in his hands his first earnings, his whole moral being undergoes a change. The money is more than money to him." It is a lively proof that he has it in his power to be "like other people" at last; to be a wage-earner, a skilled producer, a self-respecting citizen, and no miserable worthless infliction upon the charity of his friends. From the moment of his making that great discovery his industry becomes an established fact, needing restraint sometimes, but spurring rarely or never. This is the highest result that any charity can desire to produce, and it would be eminently desirable that schools doing similar work on similar lines could be established in this country.

### Whooping Cough.

Although the old habit of sending children to the gas-works to inhale the fumes which there prevail has been generally given up by the profession, there seems good reason for believing that many cases so treated did receive a certain amount of benefit from the process. The more modern substitute is the inhalation of carbolic acid or cresoline. In using the former Burney Yeo recommends that a large iron dripping-spoon should be made hot and carbolic acid vapourised by putting one or two drachms of it into the spoon. An article in the *Middlesex Hospital Journal* strongly recommends cresoline diffused by means of a cresoline vapouriser, which can be procured at any chemist, and it certainly is very efficacious in some cases. It is difficult to see why, but empiricism sometimes triumphs over disease and even over science, for we can hardly look on the air of gas works as microcidal.

